# Intra- and Inter-Regional Complexity in Multi-Channel Awake EEG Through Multivariate Multiscale Dispersion Entropy for Assessing Sleep Quality and Aging

**DOI:** 10.3390/bios15040240

**Published:** 2025-04-09

**Authors:** Ahmad Zandbagleh, Saeid Sanei, Lucía Penalba-Sánchez, Pedro Miguel Rodrigues, Mark Crook-Rumsey, Hamed Azami

**Affiliations:** 1 School of Electrical Engineering, Iran University of Science and Technology, Tehran 1684613114, Iran; a_zandbagleh@alumni.iust.ac.ir; 2 Electrical and Electronic Engineering Department, Imperial College London, London SW7 2AZ, UK; s.sanei@imperial.ac.uk; 3 Institute of Cognitive Neurology and Dementia Research (IKND), Otto-von-Guericke-University Magdeburg (OVGU), 39120 Magdeburg, Germany; lucia.penalbasanchez@med.ovgu.de; 4 Human Neurobehavioral Laboratory (HNL), Research Centre for Human Development (CEDH), Faculty of Education and Psychology, Universidade Católica Portuguesa, 4169-005 Porto, Portugal; 5 Centro de Biotecnologia e Química Fina (CBQF)—Laboratório Associado, Escola Superior de Biotecnologia, Universidade Católica Portuguesa, 4169-005 Porto, Portugal; pmrodrigues@ucp.pt; 6 UK Dementia Research Institute (UK DRI), Centre for Care Research and Technology, Imperial College London, London W1T 7NF, UK; mcrookru@ic.ac.uk; 7 Department of Basic and Clinical Neuroscience, Maurice Wohl Clinical Neuroscience Institute, King’s College London, London SE5 9RX, UK; 8 Centre for Addiction and Mental Health, University of Toronto, Toronto, ON M6J 1H1, Canada

**Keywords:** aging, EEG, machine learning, multivariate multiscale dispersion entropy, sleep quality

## Abstract

Aging and poor sleep quality are associated with altered brain dynamics, yet current electroencephalography (EEG) analyses often overlook regional complexity. This study addresses this gap by introducing a novel integration of intra- and inter-regional complexity analysis using multivariate multiscale dispersion entropy (mvMDE) from awake resting-state EEG for the first time. Moreover, assessing both intra- and inter-regional complexity provides a comprehensive perspective on the dynamic interplay between localized neural activity and its coordination across brain regions, which is essential for understanding the neural substrates of aging and sleep quality. Data from 58 participants—24 young adults (mean age = 24.7 ± 3.4) and 34 older adults (mean age = 72.9 ± 4.2)—were analyzed, with each age group further divided based on Pittsburgh Sleep Quality Index (PSQI) scores. To capture inter-regional complexity, mvMDE was applied to the most informative group of sensors, with one sensor selected from each brain region using four methods: highest average correlation, highest entropy, highest mutual information, and highest principal component loading. This targeted approach reduced computational cost and enhanced the effect sizes (ESs), particularly at large scale factors (e.g., 25) linked to delta-band activity, with the PCA-based method achieving the highest ESs (1.043 for sleep quality in older adults). Overall, we expect that both inter- and intra-regional complexity will play a pivotal role in elucidating neural mechanisms as captured by various physiological data modalities—such as EEG, magnetoencephalography, and magnetic resonance imaging—thereby offering promising insights for a range of biomedical applications.

## 1. Introduction

Sleep, a fundamental biological state of the mind and body, is crucial for maintaining cognitive function, emotional regulation, and overall physical and mental health [1,2]. Extensive evidence indicates that sleep quality is closely linked to anxiety, stress, and depression in both clinical and non-clinical populations [2]. Older adults, in particular, frequently experience disrupted sleep patterns and report poorer sleep quality compared to younger individuals [3]. However, it is essential to note that sleep problems in older adults are rarely a direct consequence of aging [4]. Consequently, the study of sleep quality has garnered increasing attention across different age groups.

Sleep quality encompasses several factors, including sleep onset latency, total sleep time, total wake time, sleep fragmentation, sleep efficiency, and disruptions such as apnea [5]. The Pittsburgh Sleep Quality Index (PSQI) is a widely utilized tool for assessing the overall sleep quality based on individuals’ retrospective evaluations of various sleep factors over the past month [6,7]. While the PSQI offers valuable insights and is a reliable indicator of perceived sleep quality, it is limited by the inherent biases associated with self-reporting and subjective measures. Subjective and objective assessments of sleep quality may not always align, with discrepancies typically more pronounced in individuals with poor sleep [8].

Electroencephalography (EEG) is a widely used, cost-effective, and noninvasive neuroimaging technique that records post-synaptic potentials from many neurons, predominantly cortical pyramidal cells [9,10,11]. Its good temporal resolution makes it particularly useful for objectively assessing sleep quality [12,13,14,15,16] and studying aging [14,17,18,19,20]. When applied during sleep, EEG frequency sub-band patterns, combined with the information from polysomnography, provide valuable insights into the sleep state and abnormalities [21]. However, sleep studies conducted in laboratory settings can be challenging. Alternatively, acquiring resting-state wake EEG presents a practical solution, allowing for data collection in a more natural setting while maintaining data quality. Growing evidence suggests that EEG complexity during wakefulness may reflect the underlying processes of sleep regulation [22,23], offering indirect insights into sleep quality without requiring overnight recordings. Although this method does not directly capture sleep-specific phenomena—such as rapid eye movement (REM) sleep or slow-wave activity (SWA)—studies have shown associations between wake EEG complexity and quantified SWA during sleep onset [22]. Consequently, combining wake EEG with self-reported sleep quality data presents a simpler yet innovative research strategy. It circumvents the challenges of traditional sleep recordings and can help assess the reliability of wake EEG in evaluating sleep quality, while preserving the benefits of lower cost, greater accessibility, and reduced participant inconvenience. Despite its potential, only a few studies [12,14,24] have explored sleep quality using awake EEG signals in healthy populations. For instance, Buckelew et al. [12] compared healthy individuals with good and poor sleep quality using resting-state and sensory/cognitive tasks. They found that theta activity decreased from the eyes-open resting state to sensory attentiveness tasks in individuals with good sleep quality, whereas the opposite trend was observed in those with poor sleep quality. Bartolacci et al. [24] assessed driving-related cognitive abilities in younger and older adults, revealing that elderly subjects exhibited lower habitual sleep efficiency and poorer performance on psychomotor vigilance, attention, and perception tasks, while younger adults reported higher self-rated sleepiness.

While power spectrum (PS) analysis, commonly used in studies examining aging and sleep, effectively identifies dominant frequency bands and linear correlations in the brain activity, it fails to capture the nonlinear and complex temporal dynamics of EEG data [25]. Nonlinear EEG processing methods have been applied separately to study either aging [17] or sleep quality [16,26]. Some of these methods include measures of statistical self-similarity, such as the Hurst Exponent (H) [26], and entropy, which quantifies the level of chaos in a dynamic system. Increased entropy and reduced interhemispheric connectivity have been observed in healthy aging, potentially indicating a decreased ability of the brain to adapt to novel or complex situations [27,28]. Jeong et al. [16] investigated eyes-closed EEG recordings from healthy male participants in both normal and sleep-deprived states. Their analysis revealed that sleep deprivation significantly reduced correlation dimension (D2), a nonlinear measure of system complexity, in specific EEG sensors. Only one study by Penalba-Sánchez et al. [14] has used nonlinear EEG measures to classify sleep quality in aging. They classified young and older individuals based on their sleep quality using nonlinear features, achieving an accuracy of 85% across all channels and up to 92% in the delta frequency band for specific channels. Their findings suggest that an algorithm combining nine complexity features effectively discriminated between age groups (e.g., young adults with good sleep quality vs. older adults with good sleep quality), particularly highlighting differences in the lower frequency bands (theta and delta). The classification accuracy improved further when comparing younger adults with good sleep to older adults with poor sleep, suggesting a relationship between aging and sleep quality at the neural level.

To further investigate these nonlinear brain dynamics, advanced analytical tools such as sample entropy (SampEn) [29] and dispersion entropy (DispEn) [30] have been developed, offering significant advantages over traditional PS analysis for EEG signal evaluation. SampEn and DispEn overcome the limitations of PS analysis by quantifying the regularity and unpredictability of EEG signals, thereby detecting nonlinear patterns that PS might overlook. Building upon these methods, multiscale entropy (MSE) [31] and multiscale dispersion entropy (MDE) [32] enhance the analysis by evaluating entropy across multiple temporal scales through a coarse-graining procedure. This multiscale approach recognizes that brain dynamics occur across various time scales—from rapid neural processing to slower network interactions—offering a more detailed and comprehensive evaluation of signal complexity [25].

MSE or MDE reveals different aspects of brain signal variability at each scale. Fine scales (short time intervals) capture local neural functions and high-frequency activities, reflecting immediate and intricate brain functions [25]. Coarse scales (longer time intervals) assess large-scale network dynamics and lower-frequency interactions, indicative of broader cognitive and integrative processes. MSE and MDE can distinguish different physiological states, such as differentiating younger from older adults, by highlighting how the complexity is distributed across these scales [32,33]. For instance, healthy EEG signals exhibit high complexity across multiple scales, suggesting robust and adaptable brain dynamics. In contrast, pathological or aging-related EEG patterns show reduced complexity, supporting the “complexity-loss” theory [31,33]. Thus, MSE and MDE provide nuanced insights into both the linear and nonlinear aspects of brain activity, making it a superior tool for understanding the multifaceted nature of EEG signals compared to conventional PS analysis [17,25].

Although MSE and MDE as single-channel entropy measures effectively evaluate the complexity and irregularity of individual EEG channels, they are limited in detecting intricate patterns and interdependencies across multiple channels [34,35]. In contrast, multivariate MSE (mvMSE) [34], and multivariate MDE (mvMDE) [34,35] leverage relationships between multiple EEG channels, enabling the identification of coordinated activity patterns that single-channel measures overlook. These multivariate approaches capture the collective dynamics of brain signals, providing a more comprehensive assessment of the data and facilitating the selection of channel subsets that better represent the underlying neural processes. While MSE and mvMSE can assess signal complexity, they may produce undefined or unreliable entropy values for short signals. In contrast, MDE and mvMDE effectively capture nonlinear dynamics and interdependencies across multiple EEG channels, resulting in higher classification accuracy or effect sizes for different health condition states [17,32,35].

A key limitation of applying mvMDE to all EEG channels is its simultaneous inclusion of both intra- and inter-regional dispersion patterns. This dual focus can obscure the analysis when the primary goal is to understand interactions between distinct brain regions, as the local complexity within regions may overshadow the interpretation of inter-regional dynamics. To address this gap, we developed and evaluated two novel strategies for optimizing mvMDE application in EEG analysis. The first strategy, namely Regional Group Analysis, involves dividing EEG sensors into five brain regions and applying mvMDE within each region to isolate intra-regional patterns. These results are then compared with the average MDE of all sensor signals within each brain region to evaluate local complexity. The second strategy, namely Representative Channel Selection, focuses on capturing inter-regional dynamics by selecting the most informative channel from each region using four feature selection methods: maximum correlation (MaxCorr), maximum entropy (MaxEn), mutual information (MI), and principal component analysis (PCA). mvMDE is then applied to these reduced channel sets, and the results are compared with traditional full-channel mvMDE and average MDE. This targeted approach helps identify key channels that capture inter-regional dynamics while reducing memory and computational demands by limiting the number of processed channels. Selecting the representative channels from each major brain region enables mvMDE to capture meaningful interactions between distinct regions, such as the frontal and temporal areas, while minimizing redundant intra-regional patterns. Together, these strategies fill an important methodological gap by improving the interpretability and efficiency of multivariate entropy analysis in EEG research.

To the best of our knowledge, no prior studies have applied multivariate entropy measures to assess sleep quality and aging, particularly using resting–awake EEG recordings. Moreover, the existing research has not explored the integration of channel selection strategies prior to conducting multivariate entropy analysis. This represents a notable gap, as channel selection can significantly reduce computational demands and potentially improve classification accuracy—especially important when working with high-density EEG data. To address these limitations, we investigated the application of mvMDE for distinguishing between younger and older adults, further subdividing each age group into good and poor sleep quality categories.

The contributions of our study are multifaceted. Firstly, we applied a multivariate multiscale entropy type and introduced a novel approach for both region-specific and representative sensor selection. Secondly, we comprehensively compare different sensor selection methods to assess the brain dynamics concerning sleep quality and aging. Both approaches exhibited trends similar to traditional methods, with slightly better results at higher scale factors (SFs), which may be related to aging. Additionally, these approaches significantly reduce computational time and memory usage, which is crucial for analyzing large datasets and high-density EEG channel recordings. Finally, this study is the first to explore both intra- and inter-regional dispersions using mvMDE, offering new insights into the brain complexity and variability across different age groups and sleep quality subgroups.

## 2. Materials and Methods

**Figure 1:**
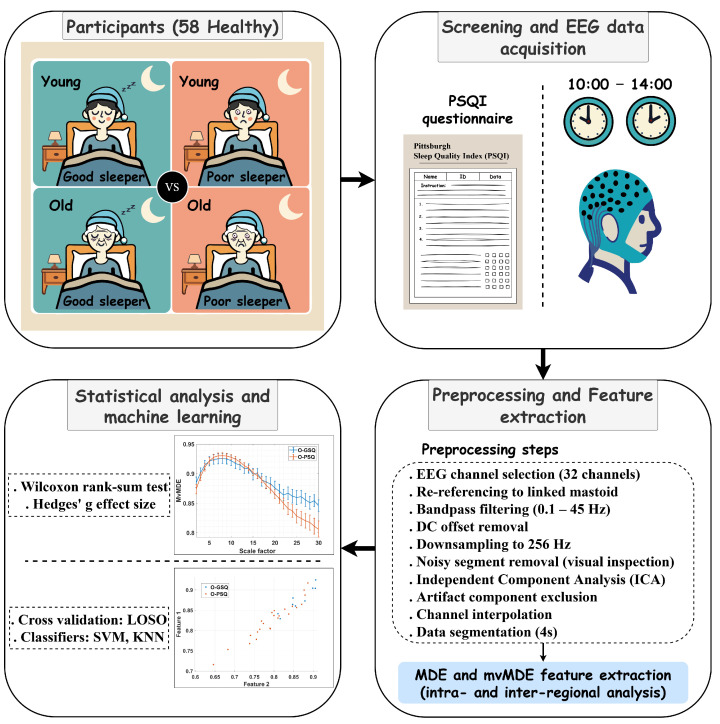
The flowchart of our study. It includes EEG data recording from four groups of healthy individuals based on PSQI scores and age, EEG preprocessing, and feature extraction using MDE and mvMDE in two innovative scenarios, followed by statistical analysis and machine learning approaches.

The flowchart of our study is shown in Figure 1. Evidently, after screening and EEG data recording from four groups of healthy individuals, the EEG data are preprocessed. The preprocessed data are then fed into our two proposed scenarios related to intra- and inter-regional analysis. Our proposed approaches include two scenarios that use entropy-based techniques, together with sensor selection. Finally, suitable statistical analysis and machine learning algorithms are applied to the extracted features to provide a comprehensive evaluation of our study. The following sections explain each of these steps in detail.

### 2.1. Participants

The current study used the eyes-closed resting-state EEG dataset reported by Penalba-Sánchez et al. [14], which received ethical approval from the Health Research Authority, UK (REC reference: 17/EM/0101). The study was conducted with 58 healthy right-handed participants, divided into the following four groups based on age and sleep quality assessed by the PSQI:

- 11 young individuals with good sleep quality (Y-GSQ) (5 females, mean age = 23.36 ± 2.70)
- 13 young individuals with poor sleep quality (Y-PSQ) (5 females, mean age = 25.53 ± 3.54)
- 9 older individuals with good sleep quality (O-GSQ) (4 females, mean age = 73.77 ± 5.45)
- 25 older individuals with poor sleep quality (O-PSQ) (17 females, mean age = 72.56 ± 3.40)

It is worth noting that sleep quality was evaluated using the self-reported PSQI questionnaire, which includes 19 items across seven subcategories: subjective sleep quality, sleep duration, sleep onset, sleep efficiency, daytime dysfunction, use of sleeping medication, and sleep disturbances [6]. This tool is considered highly effective for assessing sleep quality. The subcategories are combined into a global PSQI score, categorizing participants as good or poor sleepers in this study. Scores range from 0 to 21, with scores above 5 indicating poor sleep or with significant sleep disturbance [6]. Thus, all good sleepers have a PSQI score < 5, and all poor sleepers have a PSQI score > 5. All participants included in the study met the following inclusion criteria: they were fluent in English, had normal or corrected-to-normal vision, and fell within one of the two designated age groups—20–34 years (younger adults) or ≥65 years (older adults). Participants reported no history of cognitive, psychiatric, or neurological disorders and were not taking any medications that could affect EEG recordings. To ensure data quality and minimize potential confounding variables, participants were asked to abstain from alcohol for 24 h and from caffeine and nicotine for at least 3 h prior to the EEG recording session. Exclusion criteria included a history of paranoid or paraphrenic illness, memory impairments, dyslexia, drug abuse, or any psychiatric or neurological disorder (including current or past diagnoses) or medications that could affect EEG recordings. For the older adult group, additional exclusion criteria included a definite or probable diagnosis of Alzheimer’s disease (AD), dementia, or any dementia-related condition; a history of epilepsy or stroke; any evidence of clouded consciousness; and a history of substance abuse or dependence (including alcohol). To further screen for mild cognitive impairment (MCI), older adult participants completed the Hopkins Verbal Learning Test-Revised (HVLT-R) [36] prior to participation. This test was administered to ensure the healthy older adults were not likely to be experiencing MCI. This test was chosen due to its superior sensitivity and specificity compared to other cognitive assessments, such as the Mini-Mental State Examination (MMSE) [37]. All participants gave their informed consent before commencing the data recording.

### 2.2. EEG Data Recording and Preprocessing

The original EEG data were recorded using a BioSemi Active-Two amplifier with 128 Ag/AgCl scalp electrodes (BioSemi Inc., Amsterdam, Netherlands) at a sampling rate of 2048 Hz and digitized at 24-bits. Referencing was performed online using CMS/DRL feedback loop with a low pass filter (5th order since response with a −3 dB at 1/5th sampling rate). During the electrode application, offsets were examined to ensure they were <20 μV. In addition, seven external electrodes were placed around the eyes and face to assist with the detection and removal of ocular and facial artifacts. EEG recording took place in a specialized laboratory within a purpose-built Faraday cage to minimize electrical interference. The ambient temperature was controlled and maintained between 21–23 ∘C. Data were collected using ActiView V6.05 (National Instruments, TX, USA) on a Windows PC. Participants were seated comfortably with EEG equipment attached and were offered breaks as needed throughout the session. During data collection, they were instructed to remain still, stay awake, and keep their eyes closed throughout the resting-state EEG recording. It is worth noting that data collection was standardized between 10:00 and 14:00 to mitigate possible diurnal differences. For this study, 32 channels based on the 10–20 system (Fp1, Fp2, AF3, AF4, F3, F4, F7, F8, Fz, FC5, FC6, FC1, FC2, C3, C4, Cz, CP1, CP2, CP5, CP6, T7, T8, P3, P4, P7, P8, Pz, PO3, PO4, O1, O2, Oz) were selected, as commonly reported in studies of aging disorders [9]. This selection reduces computational load while maintaining sufficient spatial coverage for clinical and cognitive analyses. All preprocessing steps were performed using the EEGLAB toolbox [38]. After re-referencing all channels to a linked mastoid, a bandpass filter with 0.1 and 45 Hz cutoff frequencies was applied to all channels. Furthermore, the DC offset was removed, and the data were downsampled to 256 Hz. The data were then visually inspected, and all noisy segments were removed before applying independent component analysis (ICA) using the Infomax ICA algorithm. Subsequently, the components related to eye blinking or muscle artifacts were excluded, and removed channels were interpolated. Finally, the first minute of data was divided into 4-second epochs for further analysis.

### 2.3. Proposed Approaches

**Figure 2:**
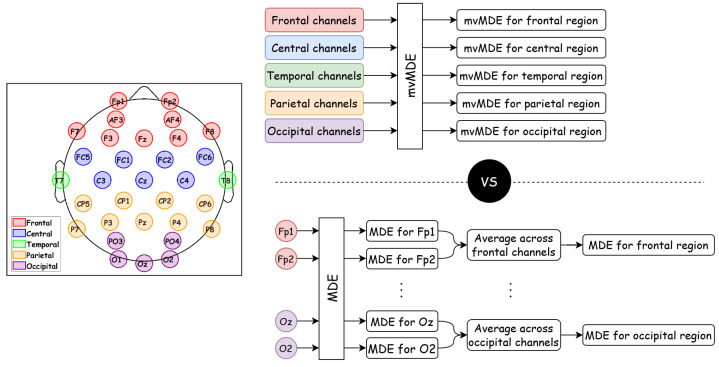
Flowchart of the first scenario, focusing on intra-regional (region-specific) analysis, demonstrating how mvMDE (top panel) and MDE (bottom panel) are compared separately for each brain region. The left topoplot illustrates the division of EEG sensors into five conventional brain regions, serving as the basis for the intra-regional analysis. Each brain region is represented by a specific color: the frontal region is shown in red, the central region in blue, the temporal region in green, the parietal region in orange, and the occipital region in purple.

In this study, we employed two scenarios reflecting intra- and inter-regional analyses to evaluate the proposed approaches comprehensively. In the first scenario, focusing on intra-regional analysis, all EEG sensors were divided into five conventional brain regions based on previous studies, as illustrated in Figure 2 (left topoplot). The proposed mvMDE was then applied separately to each regional group, yielding a complexity vector of size 1×(numberofSFs) for each region. To validate this scenario, MDE was first computed for each channel individually, and then these channel-wise metrics were compared to the mvMDE values averaged across the sensors within each region. Figure 2 provides an overview of the intra-regional analysis approach.

**Figure 3:**
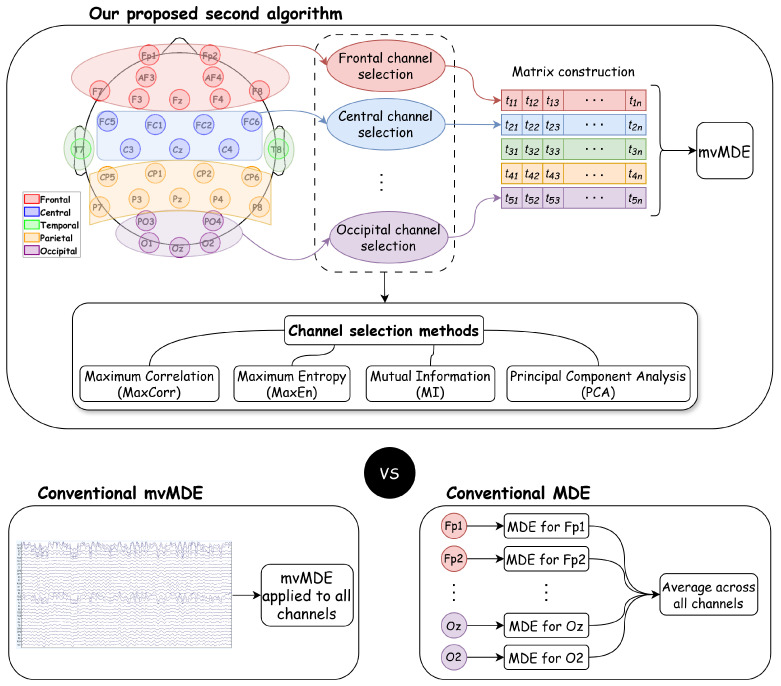
Flowchart of the second scenario, focusing on inter-regional (representative sensor selection) analysis. It illustrates the comparison between selected representative channels (via MaxCorr, MaxEn, MI, and PCA) and both the conventional MDE and the individual sensor-based mvMDE approach. Each brain region is represented by a specific color: red for the frontal region, blue for the central region, green for the temporal region, orange for the parietal region, and purple for the occipital region.

In the second scenario, centered on inter-regional analysis, we introduced an innovative representative sensor selection approach. First, one sensor was chosen from each of the five main brain regions (frontal, central, temporal, parietal, and occipital) using four different methods—MaxCorr, MaxEn, MI, and PCA. We then applied mvMDE to these selected five sensors to capture the complexity and irregularity of inter-regional EEG dynamics. The resulting complexity vector (1×(numberofSFs)) was subsequently compared with the output from both the conventional mvMDE calculation (using all 32 EEG sensors) and the averaged MDE (calculated from channel-wise MDE values). Figure 3 provides a flowchart illustrating this representative sensor selection scenario.

As can be observed, the implementation of these two scenarios required several approaches, including MDE and mvMDE calculations, as well as sensor selection methods. The following subsections present the theoretical background of these key methods, detailing their implementation and relevance to the study objectives.

### 2.4. Multiscale Dispersion Entropy

The computation of MDE involves the following steps: Consider a signal of length *L*, represented as $u=\{u_{1},u_{2},\ldots ,u_{L}\}$. First, the signal u is divided into non-overlapping segments of size τ, known as the temporal SF. The average value of each segment is then computed to produce coarse-grained signals as described in [33]:

(1) $${x_{j}}^{\left(\tau \right)}=\frac{1}{\tau }\sum_{b=(j-1)\tau +1}^{j\tau }u_{b},\;1\leq j\leq \left\lfloor \frac{L}{\tau }\right\rfloor =N$$

where *N* denotes the length of the coarse-grained series. Finally, the DispEn is calculated for each coarse-grained signal.

DispEn is a method based on Shannon entropy and dispersion patterns, offering a computationally efficient and robust approach to entropy estimation. It is particularly effective for analyzing short time series. First introduced in [30], DispEn has been extensively applied in biomedical signal analysis, including EEG [39,40], magnetoencephalography (MEG) [41], cardiac signals [42], and heart sounds [43].

The DispEn for a signal $x=\{x_{1},x_{2},\ldots ,x_{N}\}$ is computed through the following steps:

(1) Each element $x_{j}$(j=1,2,…,N) is mapped into c classes with integer indices ranging from 1 to c. This mapping is performed using the normal cumulative distribution function (NCDF) to address the problem of skewed class assignments caused by extreme values. The NCDF transforms x into $y=\{y_{1},y_{2},\ldots ,y_{N}\}$ within the interval [0, 1]:

(2) $$y_{j}=\frac{1}{\sigma \sqrt{2\pi }}\int_{-\infty }^{x_{j}}e^{\frac{-{\left(t-\mu \right)}^{2}}{2\sigma^{2}}}\;dt$$

Here, μ and σ denote the mean and standard deviation of x. A subsequent linear transformation assigns each $y_{i}$ to an integer between 1 and *c* using $z_{j}^{c}=round\left(c\cdot y_{j}+0.5\right)$, where $z_{j}^{c}$ is the $j^{\mathrm{th}}$ element of the classified signal [30].

(2) Vectors $z_{i}^{m,c}$ are constructed with an embedding dimension *m* and delay *d*:

(3) $$z_{i}^{m,c}=\left\{z_{i}^{c},z_{i+d}^{c},\ldots ,z_{i+(m-1)d}^{c}\right\},\;i=1,2,\ldots ,N-\left(m-1\right)d$$

Each vector $z_{i}^{m,c}$ is mapped to a dispersion pattern $\pi_{v_{0}v_{1}\ldots v_{m-1}}$ [30,44], where $v_{0}=z_{i}^{c}$, $v_{1}=z_{i+d}^{c}$,…, $v_{m-1}=z_{i+(m-1)d}^{c}$. The total number of possible patterns is $c^{m}$.

(3) The relative frequency of each pattern $\pi_{v_{0}\ldots v_{m-1}}$ is determined:

(4) $$p\left(\pi_{v_{0}\ldots v_{m-1}}\right)=\frac{\#\{i|i\leq N-\left(m-1\right)d,z_{i}^{m,c}\;\mathrm{matches}\;\pi_{v_{0}\ldots v_{m-1}}\}}{N-(m-1)d}$$

where # denotes the count of patterns.

(4) Finally, DispEn is computed as:

(5) $$\mathrm{DispEn}\left(x,m,c,d\right)=-\sum_{\pi =1}^{c^{m}}p\left(\pi_{v_{0}\ldots v_{m-1}}\right)\cdot \ln\left(p(\pi_{v_{0}\ldots v_{m-1}})\right)$$

### 2.5. Multivariate Multiscale Dispersion Entropy

The mvMDE algorithm is designed to extend DispEn to multivariate time series, capturing both temporal and spatial complexity. It comprises two primary steps: (1) coarse-graining the multivariate signal, and (2) calculating the multivariate dispersion entropy (mvDE). The detailed procedure is as follows:

Assume we have a multivariate time series $U={\left\{u_{k,b}\right\}}_{k=1,2,\ldots ,p}^{b=1,2,\ldots ,L}$ with *p* channels, each of length *L*.

Step 1: Coarse-Graining

For each channel *k*, the signal is divided into non-overlapping segments of size τ, termed the SF. The average of each segment is calculated to generate coarse-grained signals:

(6) $${x_{k,i}}^{\left(\tau \right)}=\frac{1}{\tau }\sum_{b=(i-1)\tau +1}^{i\tau }u_{k,b},\;1\leq i\leq \left\lfloor \frac{L}{\tau }\right\rfloor =N,\;1\leq k\leq p$$

Here, *N* is the length of the coarse-grained signal. The coarse-grained signals $X={\left\{x_{k,i}\right\}}_{k=1,2,\ldots ,p}^{i=1,2,\ldots ,N}$ represent the smoothed versions of the original multivariate time series, preserving the key dynamics at the chosen scale τ.

Step 2: Multivariate Dispersion Entropy Calculation (mvDE)

(a) Mapping to Classes: Each element $x_{k,i}$ is mapped to one of c classes with integer indices (1 to c). This mapping is achieved using the NCDF to normalize the data between 0 and 1:

(7) $$y_{k,i}=\frac{1}{\sigma_{k}\sqrt{2\pi }}\int_{-\infty }^{x_{k,i}}e^{\frac{-{\left(t-\mu_{k}\right)}^{2}}{2\sigma_{k}^{2}}}\;dt$$

where $\mu_{k}$ and $\sigma_{k}$ are the mean and standard deviation of channel *k*. A subsequent linear mapping assigns each $y_{k,i}$ to an integer between 1 and c:

(8) $$z_{k,i}^{c}=\mathrm{round}\left(c\cdot y_{k,i}+0.5\right)$$

(b) Constructing Multivariate Embedded Vectors: Based on Takens’ embedding theorem [45], multivariate embedded vectors $Z_{m}\left(j\right)$ are constructed to capture both spatial and temporal dependencies:

(9) $$Z_{m}\left(j\right)=\left\{z_{k,i}^{c},z_{k,i+d}^{c},\ldots ,z_{k,i+(m_{k}-1)d}^{c}|k=1,2,\ldots ,p\right\}$$

for j=1,2,…,N−(m−1)d, where $m_{k}$ is the embedding dimension and $d_{k}=d$ is the delay for channel *k*. For simplicity, we assume $m_{k}=m$ and $d_{k}=d$ for all *k*.

(c) Generating Combinations of Patterns: Each multivariate vector $Z_{m}\left(j\right)$ comprises $\sum_{k=1}^{p}m_{k}$ elements. For these elements, all combinations of *m* elements at a time are generated, termed $\phi_{q}\left(j\right)$, where q=1,…,mpm. The number of possible combinations for all vectors is (N−(m−1)d)·mpm.

(d) Calculating Pattern Frequencies: For each combination $\phi_{q}\left(j\right)$ and each of the $c^{m}$ possible dispersion patterns $\pi_{v_{0}\ldots v_{m-1}}$, where $v_{0}=z_{k,i}^{c}$, $v_{1}=z_{k,i+d}^{c}$,…, $v_{m-1}=z_{k,i+(m_{k}-1)d}^{c}$, the relative frequency is computed:

(10) p(πv0…vm−1)=#{j∣j≤N−(m−1)d,ϕq(j)matchesπv0…vm−1}(N−(m−1)d)·mpm

where # denotes the count of patterns in the embedded vectors.

(e) Computing mvDE: Finally, mvDE is calculated using Shannon entropy:

(11) $$mvDE\left(X,m,c,d\right)=-\sum_{\pi =1}^{c^{m}}p\left(\pi_{v_{0}\ldots v_{m-1}}\right)\cdot \ln\left(p(\pi_{v_{0}\ldots v_{m-1}})\right)$$

### 2.6. Channel Selection Methods

Channel selection in EEG signal processing is crucial for enhancing computational efficiency, minimizing overfitting, and reducing setup time in applications such as seizure detection, motor imagery classification, and emotion recognition. The primary approaches can be categorized into five strategies: filtering, wrapping, embedding, hybrid, and human-based techniques [46]. Filtering techniques independently evaluate channels using statistical measures like variance and entropy, offering speed and scalability. Wrapping techniques assess channel subsets using specific classification algorithms, achieving higher accuracy but increasing the computational complexity and overfitting risks. Embedding techniques optimize channel selection within the model training process, balancing efficiency and accuracy. Hybrid methods combine filtering and wrapping strategies, leveraging their strengths for robust performance with reduced computational demands. Human-based techniques rely on expert knowledge to identify relevant channels, which is particularly beneficial in domains requiring specialized expertise. Together, these methods provide a comprehensive toolkit for optimizing the EEG channel selection tailored to specific application needs.

In this context, it is essential to note that our goal is not to evaluate the classification results but to focus on channel selection methods’ impact on the computational efficiency and signal representation. The choice of channel selection strategy should align with the specific requirements and constraints of the application at hand without being solely dependent on the classification-based performance metrics. In this study, we propose four primary methods for channel selection: MaxCorr-based selection, MaxEn-based selection, MI-based selection, and PCA-based selection. Each technique offers unique advantages in identifying the most representative EEG channels that capture the essential characteristics of brain activity within specific regions.

#### 2.6.1. MaxCorr-Based Selection

Correlation-based selection is a method that selects EEG channels based on their average correlation with other channels within the same brain region [47]. This technique emphasizes channels that are centrally connected to the network of electrodes, thereby capturing the common activity patterns while reducing redundancy. A correlation matrix is computed for each brain region to assess the pairwise correlations between all EEG channels. The average of the absolute correlation values for each channel is then calculated. The channel with the highest average correlation is selected as the representative channel for that region. This process ensures that the chosen channel reflects the central signal of the region, maintaining essential information while minimizing overlapping activity from redundant channels. Using MaxCorr-based selection effectively identifies channels that are most representative of the collective activity within a brain region.

#### 2.6.2. MaxEn-Based Selection

Based on the principle of maximum entropy, the probability distribution that best represents the current state of knowledge about a system is the one with the largest entropy [48]. In this scenario, we select the channel with the maximum entropy value for each brain region. This approach ensures that the selected channel contains the most and least biased information, providing a comprehensive representation of the EEG signal’s complexity within that region.

#### 2.6.3. MI-Based Selection

MI measures the amount of shared information between channels, capturing both linear and nonlinear dependencies [49]. Unlike correlation, MI can identify complex relationships between EEG channels, providing a more comprehensive understanding of information sharing. The MI between every pair of EEG channels is computed for each brain region. Subsequently, the average MI with all other channels in the region is calculated for each channel. The channel with the highest average MI is selected as the representative channel for that region. This selection process ensures that the most informative and statistically relevant signals are preserved, capturing intricate dependencies that may not be evident through correlation alone. By leveraging MI for channel selection, this method captures nonlinear dependencies between EEG channels, offering a deeper insight into the underlying brain dynamics.

#### 2.6.4. PCA-Based Selection

PCA is a widely used dimensionality reduction technique that transforms the original correlated EEG channel data into a set of uncorrelated components known as principal components [50]. These components are ordered by the amount of variance they explain in the data. The primary objective of PCA in our research is to identify the EEG channel that best represents the dominant activity pattern within each brain region. For each predefined brain region, PCA is performed on the EEG data to extract principal components. The first principal component (PC1), which accounts for the maximum variance, is identified. The EEG channel with the highest loading on PC1—indicating the strongest association with the principal component—is selected as the representative channel for that region. This approach effectively reduces the complexity of the EEG data while retaining the most informative features, thereby enhancing the robustness of subsequent entropy measures. By selecting channels based on their contribution to the principal components, PCA ensures that the most significant patterns of brain activity are captured. This minimizes data dimensionality and preserves the essential information necessary for the accurate analysis of brain dynamics.

### 2.7. Statistical Analysis and Machine Learning

To rigorously evaluate our proposed algorithms, we conducted pairwise comparisons between the two groups (Y-GSQ vs. Y-PSQ and O-GSQ vs. O-PSQ) using both statistical and machine learning methods. First, the normality of the features was assessed using a one-sample Kolmogorov–Smirnov test [51]. As the features did not follow a normal distribution, the Wilcoxon rank-sum test was applied for statistical comparisons. Given the relatively small sample sizes, Hedge’s g effect size (ES) was also computed to provide a more robust evaluation and comparison of our proposed algorithms with conventional methods. In the machine learning step, we evaluated both conventional and proposed algorithms separately. Significant and informative features were first selected using the Wilcoxon rank-sum test on the training set. The test set was then assessed using two classifiers: support vector machines (SVM) and k-nearest neighbors (KNN). The selection of classifiers’ hyperparameters, as well as informative features, was optimized through a trial-and-error approach aimed at minimizing the classification error. To ensure robust and subject-independent evaluation, we performed leave-one-subject-out cross-validation (LOSOCV), where the data from one subject is used for testing while those of the remaining subjects form the training set. This process was repeated for all subjects to obtain the overall performance of each algorithm. Finally, the model performance was evaluated using specificity, sensitivity, accuracy, and F1-score metrics.

## 3. Results

### 3.1. Intra-Regional (Region-Specific) Entropy Analysis

**Figure 4:**
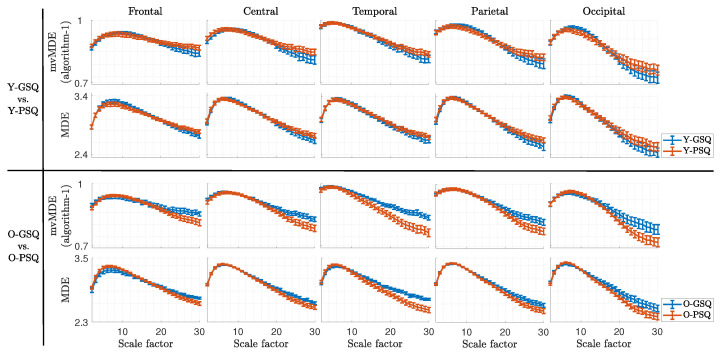
The mean and standard error of our proposed first mvMDE algorithm (first and third rows) and MDE (second and fourth rows) for comparing Y-GSQ versus Y-PSQ (top panel), as well as O-GSQ versus O-PSQ (bottom panel), across five brain regions—frontal, central, temporal, parietal, and occipital— and different SFs. In the top panel, blue lines represent Y-GSQ, and red lines represent Y-PSQ, while in the bottom panel, blue lines represent O-GSQ, and red lines represent O-PSQ.

Figure 4 illustrates the mean and standard error for both the MDE and our proposed mvMDE algorithms, comparing Y-GSQ vs. Y-PSQ and O-GSQ vs. O-PSQ across five brain regions. The first and second rows display the comparisons between the two young groups (good vs. poor sleep quality) using mvMDE and MDE, respectively, while the third and fourth rows show the same comparisons for the older groups. It should be mentioned that SFs for all multiscale measures were calculated up to 30, in order to cover the entire frequency range. Higher SFs correspond to lower frequencies, and vice versa, based on the relation that each SF (τ) corresponds to a frequency of fs/(2×τ), where fs is the sampling frequency. Thus, the full frequency range—from delta to gamma bands, each corresponding to specific SFs—was covered. Notably, the differences are more pronounced in both measures at higher SFs. Interestingly, the two paired groups exhibit opposite patterns: For instance, in the higher SFs, young adults with poor sleep quality show higher entropy than those with good sleep quality, whereas older adults with poor sleep quality demonstrate lower entropy than their counterparts with good sleep quality.

To make the comparisons more meaningful, we first determined the higher ES for each brain region using the two algorithms. Then, we statistically compared the SF corresponding to this best ES. The results of these comparisons are shown in Table 1. When comparing the two young adult groups, the best result was for our first proposed algorithm (ES = 0.677) related to the central brain region and SF = 26. It should be noted that statistically, these two groups had no significant differences (*p*-value = 0.148; z-value = −1.448) for this SF. Furthermore, when comparing the two older adult groups, the highest ES was in the temporal brain region, with SF = 27 for our proposed first algorithm (ES = 0.944; *p*-value = 0.010; z-value = 2.576). Interestingly, the best results are mostly associated with higher SF values.

### 3.2. Inter-Regional (Representative Sensor Selection) Entropy Analysis

**Figure 5:**
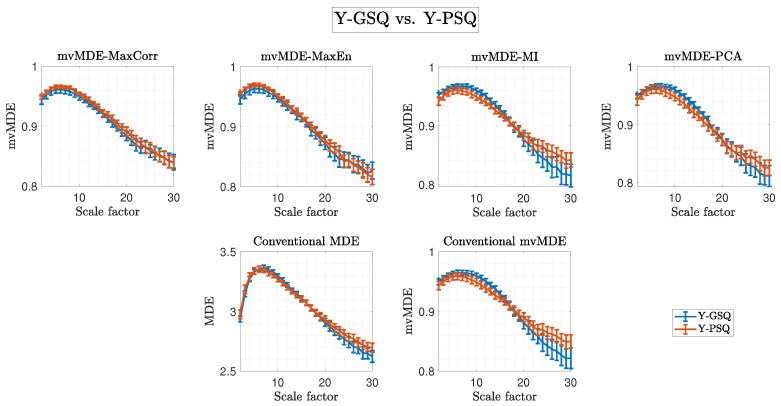
Comparison of the two groups of young adults based on the conventional MDE and mvMDE (second row) and our second proposed algorithm (first row), which is based on four methods: MaxCorr, MaxEn, MI, and PCA, for representative sensor selection across different SFs. Blue and red lines represent Y-GSQ and Y-PSQ, respectively.

**Figure 6:**
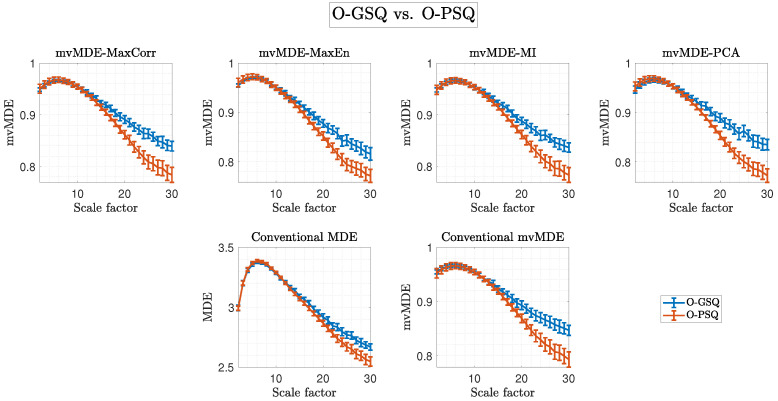
Comparison of the two groups of older adults based on the conventional MDE and mvMDE (second row) and our second proposed algorithm (first row), which is based on four methods: MaxCorr, MaxEn, MI, and PCA, for representative sensor selection across different SFs. Blue and red lines represent O-GSQ and O-PSQ, respectively.

Figure 5 presents a comparison of sleep quality in young adults using conventional MDE, full-sensor mvMDE, and our second proposed inter-regional approach, which applies four representative sensor selection methods (MaxCorr, MaxEn, MI, and PCA). Similarly, Figure 6 shows the corresponding comparisons for older adults. Notably, these results mirror the contrasting patterns observed in the intra-regional (region-specific) analysis, highlighting distinct complexity characteristics between young and older adult groups. As stated earlier, SFs for all multiscale measures were calculated up to 30, ensuring full frequency coverage.

Furthermore, Table 2 presents the SF and statistical results for the best outcomes (based on the highest Hedge’s g ES) among conventional MDE and mvMDE as well as our proposed second mvMDE algorithm when comparing Y-GSQ versus Y-PSQ and O-GSQ versus O-PSQ. It is worth noting that the highest ES values belong to the MI (SF = 11, ES = 0.670, *p*-value = 0.183, z-value = 1.333) and PCA (SF = 25, ES = 1.043, *p*-value = 0.008, z-value = 2.654) methods when comparing Y-GSQ versus Y-PSQ and O-GSQ versus O-PSQ, respectively. Additionally, our second proposed algorithm outperforms both conventional approaches, considering the highest ES values. Interestingly, the best SF values (based on the highest ES) are 25 for all approaches in older adult individuals.

### 3.3. Classification

In this study, to better evaluate our proposed algorithms and compare them with conventional methods, we used two classifiers (KNN and SVM) to differentiate between good and poor sleep in both younger and older adults. Table 3 summarizes the classification performance. The first and second rows show conventional MDE and mvMDE classification results, respectively. The third row represents region-specific mvMDE (our first algorithm), while the remaining rows correspond to our second algorithm (representative sensor selection approach).

As shown, the best results for comparing sleep quality in older adults have been achieved using the first algorithm and PCA in the second algorithm. Specifically, the first algorithm yielded an accuracy of 91.18%, sensitivity of 77.78%, specificity of 96%, and an F1-score of 0.82. Similarly, our second algorithm using PCA achieved an accuracy of 91.18%, sensitivity of 66.67%, specificity of 100%, and an F1-score of 0.80. The second-best results were obtained using MaxCorr for comparing sleep quality in older adults (accuracy = 88.24%, sensitivity = 66.67%, specificity = 96%, F1-score = 0.75).

### 3.4. Computational Time

In this study, all the processing and machine learning steps were performed using MATLAB 2024a (MathWorks, Inc., Natick, MA, USA), executed on a Windows PC equipped with 16 GB of RAM and a 2.50 GHz Intel® Core™ i5-10300H processor. For both MDE and mvMDE, we set the time lag to 1, the number of classes (*c*) to 6, the embedding dimension (*m*) to 2, and the maximum number of SF values to 30. To better evaluate our proposed algorithms, the average computational time of each function was computed for a 1-min signal. Regarding the number of channels, the computational time for both DispEn (used in the MaxEn method) and MDE was calculated for all 32 channels. Additionally, the execution time for mvMDE was measured under two conditions: using all 32 channels and using 5 channels (based on our second algorithm) to provide a more thorough evaluation. Furthermore, given the varying number of channels across brain regions, we calculated the computational time for the correlation and PCA operations using nine channels (the maximum number in the frontal region) to ensure a comprehensive assessment.

The computational times for these functions were as follows: the DispEn function required approximately 0.033 s, while the MDE function took 1.567 s for all 32 channels. For mvMDE, the execution time was 1.109 s for 5 channels and 15.208 s for all 32 channels. Regarding the sensor selection methods, the mean computation time for the MI function was 0.035 s. Additionally, when applied to nine channels, the correlation and PCA functions were executed in 0.0004 and 0.004 s, respectively.

## 4. Discussion

For the first time, this study utilizes mvMDE within two distinct algorithms—region-specific and representative sensor selection—to assess sleep quality in both young and older adults. These algorithms were statistically compared to the conventional applications of MDE and mvMDE, using ES values to compensate for the limited dataset size in each group. Additionally, each method was evaluated independently using machine learning approaches to examine classification performance.

The proposed approaches introduce significant advancements in EEG signal analysis by incorporating innovative strategies for both intra- and inter-regional complexity assessments. In the first scenario (Figure 2), mvMDE is applied to predefined EEG regions, enabling a spatially structured analysis that captures intra-regional dynamics and provides deeper insights into brain signals’ localized complexity and irregularity compared to single-channel methods. In the second scenario (Figure 3), a novel computationally efficient framework identifies representative sensors for inter-regional analysis using MaxCorr, MaxEn, MI, and PCA techniques. This ensures that selected channels retain the most critical information while substantially reducing the computational burden of processing high-density EEG datasets.

The novelty of our algorithm lies in its dual focus: enhancing interpretability through intra- and inter-regional analysis while improving scalability via efficient channel selection. This dual approach not only advances the methodological rigor of EEG analysis but also maintains or improves the accuracy of measurements, paving the way for their applications in clinical and cognitive neuroscience, especially in studies requiring the assessment of sleep quality and aging-related changes.

Our results across both scenarios demonstrate that the proposed algorithms outperform conventional approaches in distinguishing sleepers with good and poor sleep in both young and older adult groups. As shown in Figure 4, the region-specific analysis reveals that entropy measures exhibit trends similar to MDE across all brain regions. Furthermore, based on Table 1, our algorithm shows slightly better differentiation between groups, particularly at higher SF values. The highest ES for comparing older adults corresponds to SF = 27, theoretically linked to lower frequency bands, especially delta. As mentioned earlier, each SF (τ) is associated with specific frequency bands, calculated as fs/(2 ×τ), where fs is the sampling frequency. Notably, the most significant results (*p* = 0.01) in older adults occur in the temporal brain region, a critical area for studies on healthy aging and related disorders [19,52]. The hippocampus, situated in the medial temporal lobe, plays a key role in memory formation and retrieval [53], including declarative memory consolidation during slow-wave sleep [54]. The anterior hippocampus is part of an anterior-temporal network involved in semantic memory, while the posterior hippocampus is linked to a posterior-medial network associated with episodic memory [55,56]. Therefore, examining temporal lobe activity provides valuable insights into age-related memory changes and sleep disturbances. Future aging and sleep studies could combine functional magnetic resonance imaging (fMRI) and EEG to enhance spatiotemporal resolution and provide deeper insights not only into specific regions of the temporal lobe affected by aging and sleep but also into other deep brain structures, such as the locus coeruleus (LC). The LC, a brainstem region and primary source of norepinephrine, modulates wakefulness and has been identified as one of the first regions affected by both sleep processes and aging. Due to its widespread innervation of several cortical regions (e.g., strong afferent connections to the amygdala, hippocampus, and frontal areas) [57], the LC plays a critical role in regulating fear and pain responses [58], preserving memory [59], and supporting attention and decision-making [60] in both the aging process and in AD.

As illustrated in Figure 5 and Figure 6, and detailed in Table 2, the second scenario—sleep quality comparisons—further highlights the importance of higher SF values, particularly SF = 25, which is associated with the delta band and sleep disturbances in older adults. The significance of slow frequency bands (e.g., delta) has been well-documented in studies on aging [19,52,61,62,63] and sleep disorders [13,64,65]. Yeo et al. [64] recently reported significant correlations between delta and theta band powers and sleep reactivity across brain regions in older adults with insomnia, attributing these changes to aging rather than cognitive decline. Alterations in slow-frequency EEG power, such as decreased theta and delta relative band power, are associated with normal aging [19,52,61,62,63], potentially reflecting mild brain atrophy and neurophysiological changes [64,66]. Our prior findings demonstrated that decreases in slow relative power during aging align with entropy measures showing similar trends [17]. Conversely, increases in slow-frequency power and entropy have been linked to dementia and cognitive decline in older adults [17]. This study supports the hypothesis that O-PSQ is associated with reduced entropy values in the lower frequency bands (higher SFs), as evidenced by the patterns observed in Figure 4 (bottom panel) and Figure 6, which reflect a compensatory brain process aimed at maintaining cognitive function and delaying dementia progression. [67].

In contrast, as observed in Figure 4 (top panel) and Figure 5, Y-PSQ exhibit elevated entropy values in the lower frequency bands (higher SFs), suggesting a different response to sleep disturbances compared to older adults. This may stem from differences in tolerance to sleep abnormalities, such as poor sleep, forced sleep, or sleep deprivation, between young and aging brains [24,68].

Among all the methods used in the second algorithm for representative sensor selection, PCA achieves the highest ES and produces the most significant results in aging groups (Table 2). The other methods yield comparable outcomes, with slightly better performance observed when employing MI and MaxCorr for sensor selection. The classification results (Table 3) are consistent with the statistical findings, demonstrating that the second proposed algorithm, mainly when using PCA, and the first algorithm deliver the best classification performance, with SVM and KNN performing optimally, respectively. In this study we demonstrate an improvement over previous work using the same dataset in comparing O-GSQ vs. O-PSQ, achieving 91.18% accuracy with mvMDE-PCA. Notably, the earlier study [14] reported accuracies of 81% (for the best channel) and 72% (averaged across all channels). For Y-GSQ vs. Y-PSQ, their algorithm achieved 88% accuracy for the best-performing channel, but only 50% when averaged across all channels. In contrast, our approach reached a more balanced accuracy of 70.83% using mvMDE-PCA, which, although slightly lower than their best-channel performance, was derived from an integrative multichannel analysis. Despite using the same dataset, the key differences between these studies may account for the variation in the results. For instance, their model evaluated each EEG channel independently, optimizing the classification at a local (i.e., channel-specific) level. By contrast, our approach focuses on both intra- and inter-regional dynamics using mvMDE, which inherently accounts for cross-channel interactions. This methodology enables a more holistic assessment of brain complexity, better capturing the distributed nature of neural compensatory mechanisms related to aging and sleep disturbances. Overall, both studies demonstrated that changes in slow-frequency bands are characteristic of typical brain aging and poor sleep quality across the lifespan. This finding aligns with previous research using traditional methodologies, which also suggest that sleep deprivation and poor sleep quality affect theta and delta frequencies [69]. Specifically, alterations in delta and theta activity in the temporal region appear to be more strongly associated with older adults experiencing poor sleep quality, rather than with normal aging alone. We hypothesize that chronic disruptions in slow-wave activity within temporal regions in older adults may contribute to reduced clearance of brain metabolites, potentially leading to pathological aging [70]. Furthermore, studies indicate that damage in the temporal and parietal cortices driven by tau and beta-amyloid accumulation is a hallmark of prodromal AD. In particular, the right temporoparietal cortex exhibits reduced activation and increased atrophy in AD, which has been linked to impairments in spatial navigation.

In this pilot study, we evaluated our proposed algorithms, focusing on computational time and efficiency. Notably, the mvMDE function exhibited the highest computational time among all methods. However, by employing the second algorithm with only five selected sensors, we reduced the computational burden by approximately 15-fold compared to using all 32 channels. This reduction is particularly advantageous for high-density EEG recordings, making the use of multivariate entropy measures more practical for big data analysis in such settings. Furthermore, the measured MI demonstrated the shortest running time among all sensor selection methods, followed closely by PCA. Overall, combining mvMDE with the PCA-based sensor selection approach not only delivers the best classification and statistical outcomes for the aging dataset but also significantly enhances computational efficiency, making it a highly effective solution for large-scale EEG studies.

Our findings highlight the practicality of implementing the proposed approach in real-world clinical scenarios. By optimizing the computational method and utilizing conventional machine learning techniques, our method ensures easy integration into the existing clinical workflows without requiring significant computational resources. Selection of the most informative channels within each brain region further enhances efficiency, reducing computational demands while preserving analytical accuracy. Additionally, our study highlights the importance of both intra- and inter-regional approaches with multiscale analysis to identify key brain regions and frequency ranges relevant to sleep and aging-related disorders. Despite the clinical significance and applicability of this study, further validation is essential for successful clinical translation. Independent validation using diverse datasets will help assess the model’s generalizability and mitigate potential biases. Furthermore, cross-site testing across different clinical centers with varied protocols, equipment, and patient demographics will be necessary to confirm the method’s robustness and adaptability. Importantly, by employing an inter-regional approach with representative sensor selection, our method enhances the compatibility across different EEG systems, making it more accessible for use in a variety of clinical environments. These steps will be crucial for ensuring the reliability, safety, and widespread adoption of our approach in future clinical applications.

Despite the novelty of the proposed approaches and the intriguing results, this study has some limitations that should be acknowledged. First, the sample size in this study is relatively small, which may limit the robustness and generalizability of our findings. Additionally, our dataset lacks sufficient diversity in terms of demographic and ethnic representation. Future research should incorporate larger and more diverse datasets, including individuals from multiple nationalities, socioeconomic backgrounds, and age groups within each category. Expanding the sample sizes in this way would enhance the external validity of the study and reduce potential biases. Furthermore, future studies should include additional groups, particularly those related to neurodegenerative disorders such as AD and MCI, to facilitate a more comprehensive evaluation of aging and its associated conditions. Including participants with neurodegenerative disorders would provide valuable insights into the relationship between sleep quality, aging, and cognitive decline. Moreover, examining these populations could enhance our understanding of whether sleep-related biomarkers serve as early indicators of neurodegenerative conditions leading to preventative strategies and therapeutic interventions.

## 5. Conclusions

This study proposed two mvMDE-based frameworks for characterizing both intra-regional and inter-regional EEG complexity in the context of sleep quality and aging. By examining region-specific EEG sensor groupings and then employing a targeted representative sensor selection approach—leveraging MaxCorr, MaxEn, MI, and PCA—we demonstrated that these strategies enhance analytical efficiency, reduce computational overhead, and increase effect sizes. Crucially, focusing on representative sensors, particularly through the PCA-driven method, yielded the strongest discrimination of sleep quality in older adults, underscoring its clinical and research value. Moreover, the observed shifts in complexity at higher SFs tie directly into lower-frequency EEG bands, which are linked to key processes underlying sleep and aging. These insights deepen our understanding of the intricate interplay between neural complexity, cognitive function, and sleep disturbances and establish a robust methodological framework for future investigations. Beyond sleep and aging, this scalable and adaptable approach can be extended to various EEG-based applications, ranging from cognitive neuroscience and mental health assessments to the study of neurological disorders, offering a more comprehensive and efficient lens on brain complexity across diverse research domains. 

## Figures and Tables

**Table 1:** The statistical results of the highest Hedge’s g ES for MDE and our first proposed algorithm across five brain regions—frontal, central, temporal, parietal, and occipital—comparing Y-GSQ versus Y-PSQ and O-GSQ versus O-PSQ. The best results, based on the highest Hedge’s g ES, are highlighted in bold.

Group	Region	SF	MDE			mvMDE (Algorithm-1)			Best Result
			***p*** **-Value**	**z-Value**	**Hedge’s g ES**	***p*** **-Value**	**z-Value**	**Hedge’s g ES**	
Y-GSQ vs. Y-PSQ	Frontal	12	0.118	−1.564	0.629	0.817	0.232	0.284	MDE
	Central	26	0.417	0.811	0.390	**0.148**	**−1.448**	**0.677**	Proposed method
	Temporal	29	0.246	1.159	0.589	0.271	−1.101	0.521	MDE
	Parietal	30	0.164	1.390	0.571	0.385	−0.869	0.421	MDE
	Occipital	11	0.728	0.348	0.132	0.037	2.086	0.600	Proposed method
O-GSQ vs. O-PSQ	Frontal	11	0.109	1.600	0.764	0.226	−1.210	0.284	MDE
	Central	25	0.172	−1.366	0.569	0.109	1.600	0.678	Proposed method
	Temporal	27	0.021	−2.303	0.807	**0.010**	**2.576**	**0.944**	Proposed method
	Parietal	26	0.258	−1.132	0.506	0.118	1.561	0.648	Proposed method
	Occipital	24	0.114	−1.581	0.681	0.093	1.679	0.698	Proposed method

**Table 2:** The statistical results of the highest Hedge’s g ES for conventional MDE, mvMDE, and our second proposed algorithm, comparing Y-GSQ versus Y-PSQ and O-GSQ versus O-PSQ. It is worth noting that our second proposed algorithm is based on four methods: MaxCorr, MaxEn, MI, and PCA. The best results, based on the highest Hedge’s g ES, are highlighted in bold.

Group	Comparison Method	SF	MDE			mvMDE			mvMDE (Algorithm-2)			Best Result
			*p* -Value	z-Value	Hedge’s g ES	* p * -Value	z-Value	Hedge’s g ES	* p * -Value	z-Value	Hedge’s g ES	
Y-GSQ vs. Y-PSQ	MaxCorr	11	0.685	0.406	0.339	0.148	1.448	0.543	0.183	1.333	0.666	Proposed method
	MaxEn	10	0.562	0.579	0.310	0.247	1.159	0.500	0.132	1.506	0.589	Proposed method
	MI	11	0.685	0.406	0.339	0.148	1.448	0.543	**0.183**	**1.333**	**0.670**	Proposed method
	PCA	13	0.817	0.232	0.139	0.183	1.333	0.545	0.082	1.738	0.644	Proposed method
O-GSQ vs. O-PSQ	MaxCorr	25	0.138	1.483	0.598	0.056	1.913	0.771	0.008	2.654	0.880	Proposed method
	MaxEn	25	0.138	1.483	0.598	0.056	1.913	0.771	0.008	2.654	0.857	Proposed method
	MI	25	0.138	1.483	0.598	0.056	1.913	0.771	0.008	2.654	0.870	Proposed method
	PCA	25	0.138	1.483	0.598	0.056	1.913	0.771	**0.008**	**2.654**	**1.043**	Proposed method

**Table 3:** The classification performance of different EEG features using SVM and KNN classifiers for comparisons between Y-GSQ and Y-PSQ, as well as O-GSQ and O-PSQ. The best classification results are highlighted in bold.

Features	Group	Classifier	F1-Score	Specificity	Sensitivity	Accuracy
MDE	Y-GSQ vs. Y-PSQ	KNN	0.55	61.54	54.55	58.33
		SVM	0.61	61.54	63.64	62.50
	O-GSQ vs. O-PSQ	KNN	0.63	92	55.56	82.35
		SVM	0.60	80	66.67	76.47
mvMDE	Y-GSQ vs. Y-PSQ	KNN	0.55	61.54	54.55	58.33
		SVM	0.50	46.15	54.55	50
	O-GSQ vs. O-PSQ	KNN	0.59	88	55.56	79.41
		SVM	0.48	72	55.56	67.65
mvMDE (algorithm-1)	Y-GSQ vs. Y-PSQ	KNN	0.67	76.92	63.64	70.83
		SVM	0.52	53.85	54.55	54.17
	O-GSQ vs. O-PSQ	KNN	**0.82**	**96**	**77.78**	**91.18**
		SVM	0.60	80	66.67	76.47
mvMDE-Corr (algorithm-2)	Y-GSQ vs. Y-PSQ	KNN	0.59	33.33	61.54	50
		SVM	0.62	44.44	61.54	54.55
	O-GSQ vs. O-PSQ	KNN	0.75	96	66.67	88.24
		SVM	0.50	76	55.56	70.59
mvMDE-MaxEn (algorithm-2)	Y-GSQ vs. Y-PSQ	KNN	0.56	44.44	53.85	50
		SVM	0.72	66.67	69.23	68.18
	O-GSQ vs. O-PSQ	KNN	0.59	88	55.56	79.41
		SVM	0.60	80	66.67	76.47
mvMDE-MI (algorithm-2)	Y-GSQ vs. Y-PSQ	KNN	0.48	61.54	45.45	54.17
		SVM	0.60	76.92	54.55	66.67
	O-GSQ vs. O-PSQ	KNN	0.70	84	77.78	82.35
		SVM	0.53	80	55.56	73.53
mvMDE-PCA (algorithm-2)	Y-GSQ vs. Y-PSQ	KNN	0.74	53.85	90.91	70.83
		SVM	0.70	69.23	72.73	70.83
	O-GSQ vs. O-PSQ	KNN	0.78	92	77.78	88.24
		SVM	**0.80**	**100**	**66.67**	**91.18**

## Data Availability

The data are not publicly available for ethical reasons but are available from the corresponding author upon reasonable request. Additionally, the data were preprocessed using the EEGLAB toolbox (https://sccn.ucsd.edu/eeglab/index.php, accessed on 1 April 2024) in MATLAB 2024a (The MathWorks, Inc.). The processing code is available upon request from the corresponding author.

## Abbreviations

The following abbreviations are used in this manuscript:

AD	Alzheimer’s disease
DispEn	Dispersion entropy
EEG	Electroencephalography
ES	Effect size
fMRI	Functional magnetic resonance imaging
HVLT-R	Hopkins verbal learning test-revised
ICA	Independent component analysis
KNN	k-nearest neighbors
LC	Locus coeruleus
LOSOCV	Leave-one-subject-out cross-validation
MaxCorr	Maximum correlation
MaxEn	Maximum entropy
MCI	Mild cognitive impairment
MDE	Multiscale dispersion entropy
MEG	Magnetoencephalography
MI	Mutual information
MMSE	Mini-mental state examination
MSE	Multiscale entropy
mvDE	Multivariate dispersion entropy
mvMDE	Multivariate multiscale dispersion entropy
mvMSE	Multivariate multiscale entropy
NCDF	Normal cumulative distribution function
O-GSQ	Old individuals with good sleep quality
O-PSQ	Old individuals with poor sleep quality
PCA	Principal component analysis
PS	Power spectrum
PSQI	Pittsburgh sleep quality index
REM	Rapid eye movement
SampEn	Sample entropy
SF	Scale factor
SVM	Support vector machine
SWA	Slow-wave sleep
Y-GSQ	Young individuals with good sleep quality
Y-PSQ	Young individuals with poor sleep quality
